# The impact of COVID-19 on the intention of third-child in China: an empirical analysis based on survey data

**DOI:** 10.1186/s12889-023-15944-w

**Published:** 2023-06-21

**Authors:** Zi Li, Siwen Qian

**Affiliations:** 1 Department of Public Administration, Chongqing Technology and Business University, Chongqing, China; 2 Department of Law and Sociology, Chongqing Technology and Business University, Chongqing, China

**Keywords:** COVID-19, Fertility, Intention, Logit model, Mediation analysis, China

## Abstract

**Background:**

Against the grim background of declining intention to have children, the ravages of COVID-19 have pushed China and the world into a more complex social environment. To adapt to the new situation, the Chinese government implemented the three-child policy in 2021.

**Objective:**

COVID-19 pandemic indirectly affects the country's internal economic development, employment, fertility plans or intention, and other major issues related to the people's livelihood, while undermining the stable operation of society. This paper explores the question that will COVID-19 pandemic affect Chinese people's intention to have a third child. And What are the relevant factors inside?

**Method:**

The data in this paper are from the Survey released by the Population Policy and Development Research Center of Chongqing Technology and Business University (PDPR-CTBU), including 10,323 samples from mainland China. This paper uses the logit regression model and KHB mediated effect model (a binary response model given by Karlson, Holm, and Breen) to investigate the impact of the COVID-19 pandemic and other factors on Chinese residents' intention to have a third child.

**Results:**

The results suggest that the COVID-19 pandemic has a negative effect on Chinese residents' intention to have a third child. In-depth research on the mediating effect of KHB shows that COVID-19 pandemic will further inhibit residents' intention to have a third child by affecting their childcare arrangements, increasing their childcare costs, and increasing their exposure to occupational hazards.

**Contribution:**

This paper is more pioneering in focusing on the impact of the COVID-19 epidemic on the intention to have three children in China. The study provides empirical evidence for understanding the impact of COVID-19 epidemic on fertility intentions, albeit in the context of policy support.

## Background

Against the grim background of a declining intention to have children, the ravages of COVID-19 have pushed China and the world into a more complex social environment [1–3]. With the continuous transformation of society, the demographic dividend has gradually reduced the impetus, and the population structure of aging and fewer children has gradually emerged. The one-child policy, which encourages couples to have one child per couple, is no longer compatible with social reality [4]. In this reality, China has taken the step of progressively liberalizing its fertility policy restrictions to raise the fertility rate and avoid a fertility trap. At the niIn years from 2013 to 2021, China's birth policy has made a major adjustment from "only two children" to "universal two children" [5, 6]. In May 2021, the Central Committee of the Communist Party of China (CPC) issued the Decision on Optimizing family Planning Policy to Promote Long-term Balanced Development of Population, which indicates that China has officially entered the era of a universal three-child policy [7–9].

Before the opening of the two-child policy, the fertility intentions of Chinese residents were generally restricted by the policy. After the opening of the two-child policy, the rate of decline of China's total population and labor force population has been slowed down somewhat [10, 11], but the actual fertility level of residents is still significantly inferior to the policy fertility level [5]. Therefore, the effect of liberalizing the policy limit again is yet to be considered. With the continued impact of a major social health event such as COVID-19, the issue of fertility rebound after the opening of the three-child fertility policy has become an even more important area of scholarly and national concern. Previous studies have shown that the impact of COVID-19 pandemic on fertility depends on the development model of the corresponding society and the stage of demographic transition [12–16]. And the emergence of COVID-19 made China's fertility level "worse" and the number of births continued to decline [17]. Economic and social changes caused by COVID-19 pandemic may also affect fertility intention and behavior.

Although fertility intentions sometimes do not directly translate into actual fertility behavior, understanding and exploring the feedback on fertility intentions at the early stage of the policy (less than one year after the three-child policy was introduced at the time of data collection) is important for us to anticipate the likely change in fertility rates [18, 19]. Based on previous policy feedback, it is possible to speculate that the three-child policy will likely not have much of a significant impact on fertility levels and fertility in China (it is, after all, somewhat targeted) [20–22], but it is important to understand whether, and to what extent, and how, it is affected by low intentions due to the COVID-19. This can provide a useful basis for understanding the social impact of major health events in China and around the world [23], and for anticipating the likely changes in fertility in the post-epidemic era.

Therefore, the most important research objective of this paper is to investigate whether COVID-19 affects people's willingness to have three children in the early stage of the opening of the three-child policy, to further discuss through what the COVID-19 mainly has an impact.

## Methods

### Data sources

**Fig. 1:**
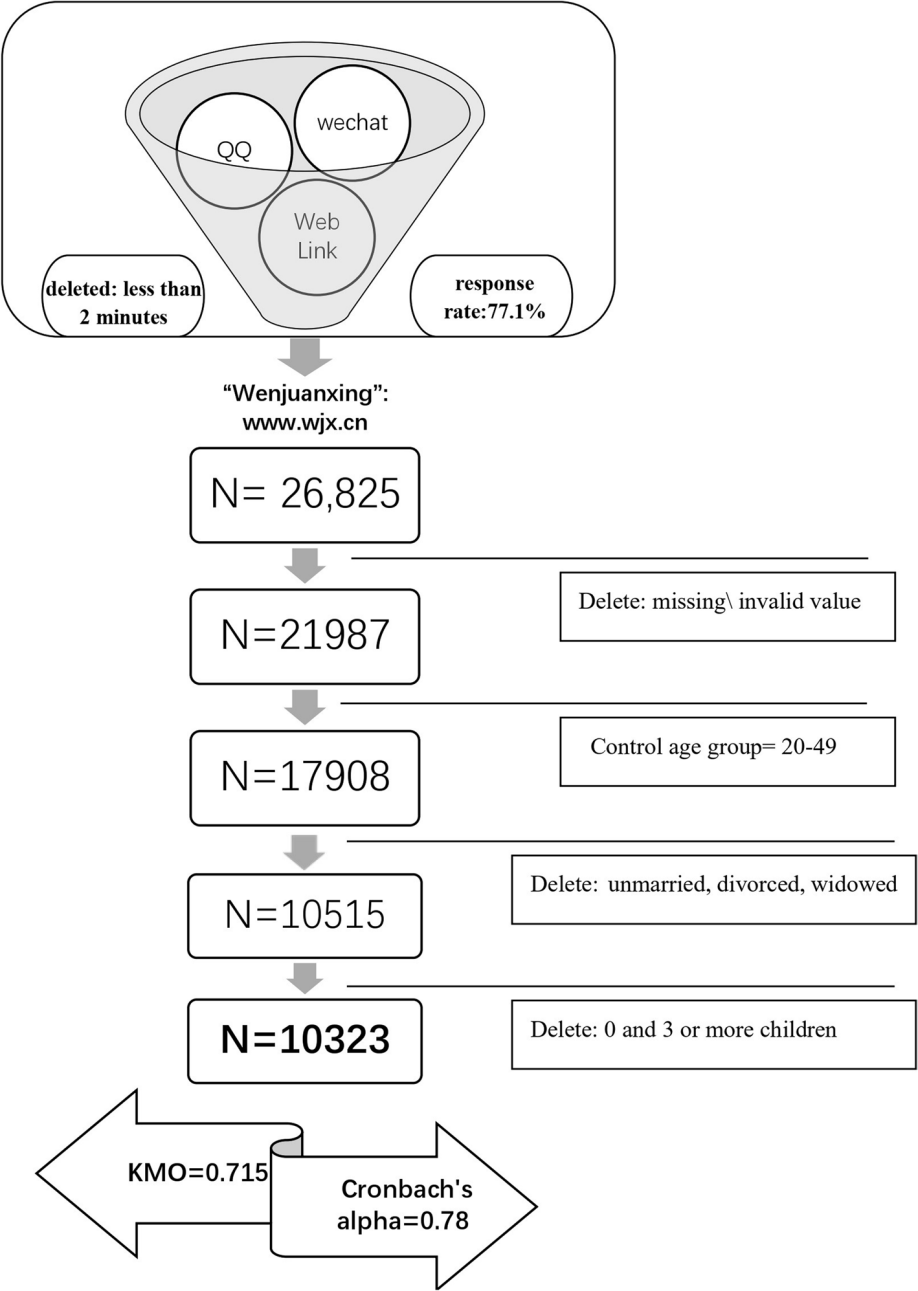
Data sources and the filtering process

The data sources and the filtering process in this paper are shown in Fig. 1 below. The data in this paper are from the Survey on Marriage and Childbearing Behavior in the Digital Era released by PDPR-CTBU (hereinafter referred to as the survey). To explore the impact of COVID-19 pandemic on human fertility, parenting and, education behaviors, and to understand the fertility intention after the universal three-child policy, PDPR-CTBU selected the online survey platform called “Wenjuanxing”: www.wjx.cn, the dominating online survey platform in China, for the online questionnaire. Then through the mainstream social media platforms in China like WeChat and QQ to promote [24–29]. This is mainly because the studies on fertility intentions under the simultaneous occurrence of the three-child policy and COVID-19 have certain critical time limits, with which data collection must strictly comply with the national prevention and control policy [30].

The questionnaire webpage received a total of 34,789 hits in the three months since it was sent out to the closing date of the survey. According to the analysis of IP addresses filled by users, 96.73% of questionnaires were filled by mobile clients. The regions involved in the country's 29 provincial administrative regions of China, accounted for 85.3% of all. The survey was voluntary and anonymous, and participants were allowed to exit the questionnaires at any time if they were still concerned about information leakage, and such questionnaires would not be submitted. According to Revilla & Ochoa et al. [31, 32], too short a response time online will affect the quality of the questionnaire, so we deleted the data of users who filled with less than 2 min of response time. In the end, 26,825 valid and complete questionnaires were obtained, with a response rate of about 77.1%.

### Data inspection and preprocessing

The basic purpose of a sample survey is to obtain information about the sample using probability sampling and to infer the parameters of the total from it. Therefore, only the indicators obtained after the weighting process can represent the overall level [33, 34]. To correct the bias of online survey data and ensure data quality, this paper follows the practices of Yaer. Z and Demery et al. [35, 36]. to correct the demographic characteristics such as "gender structure", "age structure", and "marital status" on the survey data according to the structural characteristics of the seventh Chinese census data. Identify the target group as the 20–49-year-old age group. Considering that fertility behavior in China needs to occur when one is married to a living partner, we removed the unmarried, divorced, and widowed samples from the variables, for a total of 640. At the same time, it is not appropriate to ask a family about their desire to have three children when they do not currently have any child. Since the inquiry of willingness to have more children should be based on the fact that one has already had children, we removed the sample of married people who do not have children yet. Only the sample with one or two children is discussed.

Then, this paper removes the missing value, invalid value, and meaningless value of relevant variables. Because this paper studies the intention to have three children, to avoid self-selection bias, then deleted the samples that already has three or more children before the outbreak. Finally, a total of 10,323 samples are input into Stata software for data quality testing.

To further verify the data, this paper reviewed it by using Cronbach's alpha and the KMO test. Cronbach's alpha measures the internal consistency, or reliability, of a set of survey items, that quantifies the level of agreement on a standardized 0 to 1 scale. Higher values indicate higher agreement between items [37]. The Kaiser–Meyer–Olkin (KMO) Test is a statistical measure to determine how suited data is for factor analysis, and the criterion is calculated and returns values between 0 and 1 [38]. When the KMO value is closer to 1, it means that the correlation between variables is stronger and the original variables are more suitable for factor analysis.

The value for Cronbach’s Alpha for the survey data was α = 0.78. The KMO test value between related variables is greater than 0.5 (0.715). That means the reliability and validity test of the questionnaire is passed. The following Table 1 describes how different values of Cronbach’s Alpha are usually interpreted:

**Table 1:** Information on the value of Cronbach's Alpha

Cronbach’s Alpha	Internal consistency
0.9 ≤ α	Excellent
0.8 ≤ α < 0.9	Good
0.7 ≤ α < 0.8	Acceptable
0.6 ≤ α < 0.7	Questionable
0.5 ≤ α < 0.6	Poor
α < 0.5	Unacceptable

### Variable selection and descriptive statistics

This paper aims to explore whether the outbreak of COVID-19 pandemic and the extent of its impact on individuals will affect people's intention to have three children in the era of China's universal three-child policy, and what influencing factors are involved. Here, some important variables involved in empirical research and the basis for assigning values will be explained.

#### Dependent variable

The core explanatory variables of this paper come from the questionnaire question: Do you have the intention to have three children? Assign the answer to yes = 1; No = 0, thus forming a dichotomous variable.

#### Independent variables

Three explanatory variables are selected in this paper, including COVID-impact, gender, and the number of existing children.

**Fig. 2:**
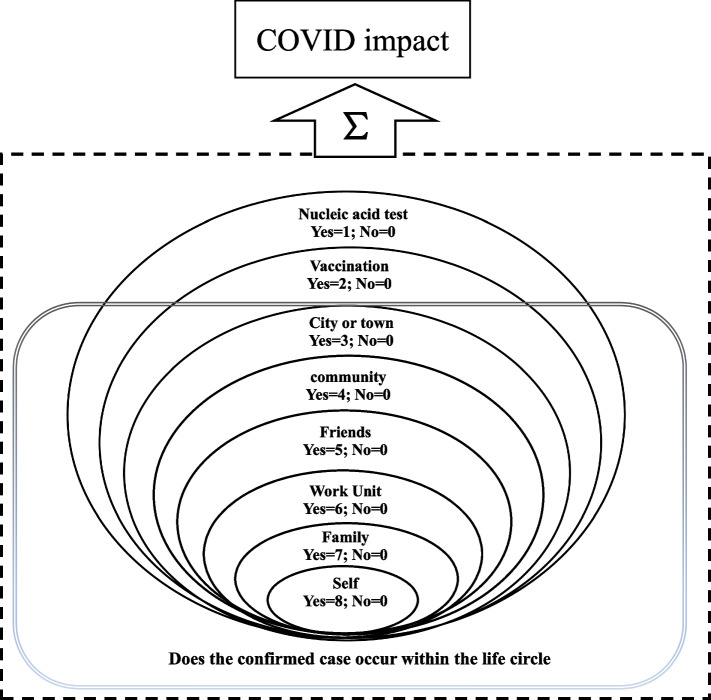
COVID-19 impact definition logic

According to the concept of life circle, the area where the inhabitants actually live, a circle is formed between the central area and the surrounding areas according to the will of self-development and the conclusion of agreements, which is commonly used in the field of geography and planning research [39]. When a confirmed case is present in a person's life circle, the degree of impact on their lives will increase significantly [3]. In the questionnaire design, questions about the impact of COVID-19 are graded according to whether there are confirmed cases in people's living areas from near to far, in accordance with China's rules of comprehensive pandemic monitoring and key containment. In addition, weight assignment is carried out based on the impact level to form the key explanatory variable: COVID-19 impact. The variable definition logic is shown in Fig. 2 below.

And its calculation formula is as follows,

1 $$COVID\;impact=\sum_i^8B_{i}$$

where B represents the COVID-19 variables in the questionnaire and $i$ represents the labels of relative variables. In the final statistics, the lowest score for residents is 0, and the highest score is 36. There is a large difference in COVID-19 impact scores between individuals.

The number of existing children is intended to explore whether having different numbers of children will affect people's intention to have a third child.

#### Control variables

To reduce the interference of confounding variables on model estimation, we include 6 control variables that have been identified in the literature as being related to fertility intention, including age, residence type, hukou type, education level, employment status, and monthly income level [9, 40]. Among them, the type of residence and the type of hukou are different concepts. Due to the legacy of some historical policies, China still retains a dualistic registration structure, where people are divided into two types of the hukou structure system, agricultural registration and non-agricultural registration. For residence type, it is a type of the area where the person is actually living. There are large migrant population group in China, some of which may, for example, belong to an agricultural household but do not live in rural communities (or vice versa). So the researchers in China have tried to ask respondents about their hukou type and their residence type in the survey, so that they can determine to some extent whether people belong to the migrant population [41, 42].

#### Intermediate variables

Three mediator variables were selected to test the mediating effect, namely, childcare arrangement, childcare cost, and occupational virus exposure. The childcare arrangement variable comes from the questionnaire question: whether your child's care arrangement has been affected during the COVID-19 outbreak. Assign the answer of yes = 1; no = 0, so a dichotomous variable is formed. The childcare cost variable is derived from the questionnaire question: whether your childcare costs have increased during the COVID-19 pandemic. Likewise, dichotomous variables are formed based on the responses.

The “occupational exposure to COVID-19” variable is taken from the answers to the questionnaire "A7—Current Employment field". In the recall data for this question, we divided the respondents' occupations into 20 categories according to their fields. During COVID-19, to ensure the normal functioning of residents' daily lives. Some occupational groups must work continuously in an environment that is infinitely close to the risk of virus transmission [43–45], such as healthcare workers, material transportation and supply workers, community workers, etc. Then, this research combined with Guidelines for the prevention and control of COVID-19 in key places, key units, and key groups (Joint Prevention and Control Mechanism Comprehensive Development [2021] 82) released by the National Health Commission of China and related research [46–48] divided these 20 occupational types are into three types of occupational exposure, which are high, medium and low exposure, and the classification variable of occupational virus exposure is respectively assigned according to the weights (Table 2).

### Statistical analysis

**Fig. 3:**
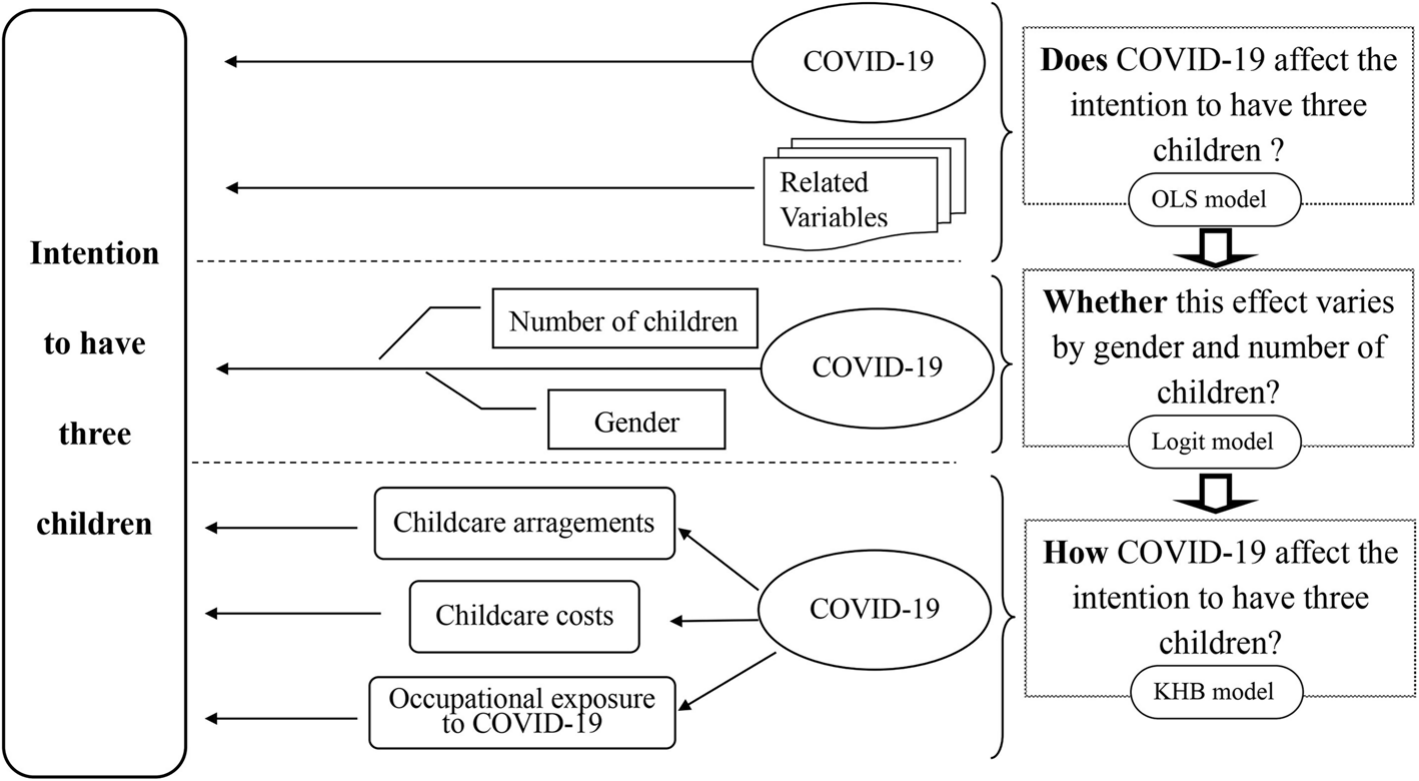
Path and structure of empirical research

Referring to the recency determination theory of fertility rate and previous research results, this paper uses the obtained empirical sample data to construct the research framework (Fig. 3). Moreover, Stata17.0 software is adopted to conduct an empirical analysis of the data.

In this paper, a total of three research methods are selected to conduct the study, which are the OLS model logit model and the KHB mediated effect model.

The OLS model was first applied to perform a basic linear regression of the intention to have three children. Since the linear probability model does not apply to binary discrete dependent variables, scholars usually use Logit and Probit analysis models in empirical studies on dichotomous variables (e.g., fertility intention). This paper selects binary Logit regression as the benchmark regression model for analysis. Logit mode is one of the discrete selection method models, and its model formula is as follows,

2 $$p(Y=1\vert\chi\;=x)\;=\frac{\exp\left(x'\mathrm\beta\right)}{1-\exp\left(x\mathit'\beta\right)}\;$$

Due to the scale problem, the mediator variable detection technique of fitting the mediation model cannot be applied to the nonlinear regression model. Kohler, Karlson, and Holm developed the "confounding effect" and "scale change effect" in recent years, also known as KHB technology, which can solve the above scale problems and effectively estimate the mediator variables of the nonlinear regression model [49]. The KHB model measures total, direct, and indirect effects through the logit method [50]. Total effects can be decomposed into direct and indirect effects by comparing the coefficients of the linear model [51]. The basic idea was to compare the full model with a reduced model that substitutes (for the mediator variable) the residuals of the mediator from a regression of the mediator on the key variables. This method allows the separation of the change in the coefficient due to either confounding or rescaling [52, 53]. Three variables ${Z}_{1}$ childcare arrangement, ${Z}_{2}$ childcare cost and ${Z}_{3}$ occupational virus exposure are included in the regression model as mediator variables, and KHB method is used to detect the mediating effect. KHB method is mainly calculated by incorporating residual R into the regression equation (Eq. 5), and this residual R is the regression residual result of confounder Z and X (R and X are orthogonal).

3 $$Y^\ast=\widetilde{\infty_{R}}+\widetilde{\beta_{R}} X+\widetilde{\gamma_{R}}R+\delta_RC+\varepsilon$$

Because of ÓR=ÓF，βR=βF, the following formula (4) is obtained. Finally, the mediating effect is divided by a common scale, and the model analysis results are obtained.

4 $$\widetilde{b_R}-b_F=\frac{\widehat{\beta_R}}{\widetilde{\sigma_R}}-\frac{\beta_F}{\sigma_F}=\frac{\beta_R-\beta_F}{\sigma_F}$$

## Results

### Descriptive statistics

Table 2 shows the statistics of the main variables in this paper. According to descriptive statistics, 655 respondents have an intention to have three children, accounting for only 6.3% of the total, and 93.7% of respondents said they do not have or have no idea when they will have a third child. The majority (63.4%) of respondents who were willing to have three children said they did not yet have a clear plan for having a third child, while only 23.9% planned to implement a three-child birth plan within two years. 78.5% of the respondents are female, and those with one, and two children account for 68.91%, and 31.04% of the total, respectively.

**Table 2:** Main variable statistics

Variable	Obs	Definition& assignment	Mean	Std. Dev
Intention to have three children	10,323	0.NO 1. YES	.063	.243
COVID-19 Impact	10,323	The degree to which individuals are affected by the COVID-19(range:0–36)	3.78	2.39
Gender	10,323	0 = male 21 = female	.785	.411
Number of existing children	10,323	1 = one child 2 = two child	1.331	.495
age	10,323	Numerical value	37.819	6.135
Type of residence	10,323	0 = Urban committee 1 = Rural committee	0.339	0.473
Type of hukou	10,323	0 = Agricultural registration 1 = Non-agricultural registration	0.329	0.47
Education level	10,323	1 = No education 2 = Pre-school 3 = Primary school 4 = Middle School 5 = High School 6 = University 7 = Undergraduate 8 = Master 9 = Doctoral	5.997	1.142
Employment Status	10,323	1 = Staying at home before and after the epidemic 2 = Unemployed due to the epidemic 3 = Continuing to work in the same unit 4 = Employed but changed workplace	3.113	.316
Income per month	10,323	1 = No income 2 = Under 2000 RMB 3 = 2001-5000RMB 4 = 5001–10,000 yuan 5 = 10,000 or more than 20,000 yuan or less 6 = 20,000 yuan or more than 50,000 yuan or less 7 = 50,000 yuan or more than 100,000 yuan or less 8 = 100,000 yuan or more	3.218	.92
COVID-19-child care arrangements	10,323	Whether COVID-19 affected personal childcare arrangements 0 = NO 1 = YES	.424	.494
COVID-19- childcare cost	10,323	Has COVID-19 increased personal childcare costs 0 = NO 1 = YES	.436	.496
Occupational exposure to COVID-19	10,323	Exposure of individuals to the virus in their occupations during the COVID-19 3 = High exposure occupations	1.934	.798
		2 = Medium exposure occupations		
		1 = Low exposure occupations		

In terms of the impact of COVID-19, only 8.89% of the respondents statistically felt that their was distant from COVID-19, and they not only didn’t perceive the presence of confirmed cases within their lives during the outbreak, but also did not have nucleic acid testing or vaccination. A high degree of perception was found by 34.28% of the respondents, who believed that COVID-19 was already around them and was seriously affecting their personal lives. The mean COVID-19 Impact value of 3.78 means that, overall, people generally believe that COVID-19 has a significant impact on them and has limited their range of activities to a certain extent.

42.5% of respondents thought their childcare arrangements had been affected by COVID-19. And 43.6% of respondents believe that the epidemic has increased their childcare costs. After categorizing the respondents according to their occupational field, we found that people who belonged to high-risk occupations during the outbreak period accounted for 28.8% of the total, medium-risk occupations accounted for 36.6%, and low-risk occupations accounted for 35.14%., and the overall distribution is relatively balanced.

### Factors that affect the intention to have three children

According to Table 3 below, the results of the OLS linear regression model showed that there were many factors that influenced residents to have a third child, including the impact of COVID-19 (*p* < 0.05), gender (*p* < 0.01), number of children (*p* < 0.01), and age (*p* < 0.05). Among them, only the number of children variable was positively affected, i.e., the more existing children they have, the more willing to have three children. In order to avoid covariance, this paper uses the variance inflation factor (VIF) to assess its covariance, and the results show that the mean VIF = 1.31, which is much smaller than 10, proves that there is no covariance in the model.

**Table 3:** OLS and Logit Model of the COVID-19 on the intention to have three children

	**Y = Intention to have three children**									
	**OLS model**		**Logit model**							
	**underlying linear**		**No control variables**				**With control variables**			
	**Model 1**		**Model 2**				**Model 3**			
	coef	SE	coef	Z	dy/dx	SE	coef	Z	dy/dx	SE
COVID-19 Impact	-0.002**	-0.001	-0.428***	-0.058	-0.022***	-0.003	-0.402***	-0.059	-0.021***	-0.003
Gender	-0.042***	-0.006								
Gender *COVID			-0.143***	-0.02	-0.007***	-0.001	-0.143***	-0.02	-0.007***	-0.001
Number of children	0.050***	-0.005								
Number of children *COVID			0.187***	-0.02	0.010***	-0.001	0.180***	-0.02	0.009***	-0.001
Type of residence	0.008	-0.006					0.167*	-0.099	0.009	-0.005
Type of household	0.005	-0.006					0.139	-0.1	0.007	-0.005
Education level	0.003	-0.002					0.054	-0.046	0.003	-0.002
Employment Status	-0.002	-0.006					-0.048	-0.121	-0.002	-0.006
Income per month	0	0					-0.002	-0.007	0	0
age	-0.001**	0					-0.02	-0.05	-0.001	-0.002
Constant	0.057*		-2.518***				-2.049***			
	(-0.032)		(-0.075)				(-0.584)			
chi2			530.48***				495.51***			
Pseudo R2	0.02		0.033				0.085			
Akaike information criterion (AIC)			6000.828				5992.06			
The area under the curve(AUC)			0.797				0.799			
**Observations**	10,323		10,323				10,323			

(1) *** *p* < 0.01, ** *p* < 0.05, * *p* < 0.1; (2) AIC = (2*K-2*LL)/n, in which, LL is the likelihood, K is the number of predictors, and n is the number of observations

Based on the basic linear regression, the logit model is chosen to further investigate whether the COVID-19 negatively affects the intention to have three children differently depending on gender and the number of children, and the interaction term between the them and the COVID-19 impact is included in the model for calculation, and the models with and without control variables are shown separately for comparison.

To ensure the robustness of the conclusion, both the Probit and logit models are used in this paper for estimation. As indicated by the results (Table 4 below), the sign direction and significance of the results estimated by the Probit model and the Logit regression method are consistent. The AUC values for both models are the same and both are very close to 0.8 (0.797&0.799), which means they all have a better model performance. But compared to the Probit model, the Logit model has the smallest AIC score (5992.060) and its accuracy of it is 93.88% which represents a better model fit. In conclusion, this paper is robust in the estimation results of the Logit model.

**Table 4:** Logit and Probit Model of the COVID-19 on the intention to have three children

	**Logit model**				**Probit model**			
	**No control variables Model 2**		**With control variables Model 3**		**No control variables Model 4**		**With control variables Model 5**	
VARIABLES	coef	dy/dx	coef	dy/dx	coef	dy/dx	coef	dy/dx
COVID-19 Impact	-0.428***	-0.022***	-0.402***	-0.021***	-0.186***	-0.020***	-0.174***	-0.019***
	(0.058)	(0.003)	(0.059)	(0.003)	(0.028)	(0.003)	(0.028)	(0.003)
Gender *COVID	-0.143***	-0.007***	-0.143***	-0.007***	-0.067***	-0.007***	-0.067***	-0.007***
	(0.020)	(0.001)	(0.020)	(0.001)	(0.010)	(0.001)	(0.010)	(0.001)
Number of children *COVID	0.187***	0.010***	0.180***	0.009***	0.085***	0.009***	0.082***	0.009***
	(0.020)	(0.001)	(0.020)	(0.001)	(0.010)	(0.001)	(0.010)	(0.001)
Type of residence			0.167*	0.009			0.083*	0.009*
			(0.099)	(0.005)			(0.047)	(0.005)
Type of hukou			0.139	0.007			0.063	0.007
			(0.100)	(0.005)			(0.049)	(0.006)
Education level			0.054	0.003			0.025	0.003
			(0.046)	(0.002)			(0.021)	(0.002)
Employment Status			-0.048	-0.002			-0.023	-0.003
			(0.121)	(0.006)			(0.058)	(0.006)
Income per month			-0.002	-0.000			-0.000	-0.000
			(0.007)	(0.000)			(0.003)	(0.000)
age			-0.020	-0.001			-0.012	-0.001
			(0.050)	(0.002)			(0.023)	(0.002)
Constant	-2.518*** (0.075)		-2.049*** (0.584)		-1.469*** (0.040)		-1.250*** (0.277)	
chi2	530.48***		495.51***		451.73***		461.99***	
Pseudo R2	0.033		0.085		0.033		0.081	
Akaike information criterion (AIC)	6000.828		5992.060		6020.273		6013.637	
The area under the curve(AUC)	0.797		0.799		0.797		0.799	

(1) When dy/dx is reported robust standard errors in parentheses;(2) When coef. is reported, the Z-value in parentheses;(3) *** *p* < 0.01, ** *p* < 0.05, * *p* < 0.1; (4) AIC = (2*K-2*LL)/n, in which, LL is the likelihood, K is the number of predictors, and n is the number of observations

As depicted in Table 3, whether control variables are included or not, the influence of COVID-19 ($\beta$ =-0.428; *p* < 0.01) are negatively significant to the intention to have a third child within. that is, the greater the impact of COVID-19 on individuals, the lower their intention to have a third child. According to the probability ratio, the intention of residents to have a third child decreases by 2.2% for every one-point increase in the impact of COVID-19.

In addition, the number of children ($\beta$ =0.05; *p* < 0.01) and the gender ($\beta$ =-0.04; *p* < 0.01) themselves have an impact on the intention to have three children. That is, those who have two children are more willing to have three children than those who have only one child, and women are more reluctant to have three children than men.

Also, the depression of the willingness to have three children by COVID-19 varies by gender ($\beta$ =-0.14; *p* < 0.01) and number of children ($\beta$ =0.18; *p* < 0.01). Under the influence of the COVID-19, women showed more reluctance to have three children. And families with two children seemly had less fluctuation in their willingness to having another children due to the COVID-19 compared to families with only one.

At present, there are many studies on fertility intention and its factors worldwide, whereas few studies on how COVID-19 affects people's fertility intention from the perspective of major public health emergencies. Therefore, this paper will further explore its internal mechanism through the mediating effect to fill relevant research gaps to some extent.

### The mediating effect of the COVID-19 pandemic on third-child fertility intention

According to the analysis above, it has been concluded in this paper that COVID-19 pandemic has a certain inhibiting effect on Chinese residents' intention to have a third child. On this basis, the total, direct and indirect effects of the three variables were calculated separately using the KHB model in the logit model of COVID-19 affecting fertility intention. Variables such as gender and number of children were also controlled for in the model. The results are shown in Table 5 below.

**Table 5:** Mediating effects of COVID-19 on the intention to have three children

Effect	β	SE	Mediation (%)
**Model 1: COVID-19 → Childcare arrangement → intention to have three child**			
Total effect	-0.092***	-0.019	
Direct effect	-0.086***	-0.019	
Indirect effect	-0.006*	-0.003	**6.5**
**Model 2: COVID-19 → Childcare cost → intention to have three child**			
Total effect	-0.101***	-0.019	
Direct effect	-0.095***	-0.019	
Indirect effect	-0.006***	-0.002	**5.9**
**Model 3: COVID-19 → Occupational exposure → intention to have three child**			
Total effect	-0.092***	-0.019	
Direct effect	-0.090***	-0.019	
Indirect effect	-0.002**	-0.001	**2.2**

(1) *** *p* < 0.01, ** *p* < 0.05, * *p* < 0.1;

As shown in model 1, COVID-19 had negative indirect effects on the intention of three children through affecting residents’ childcare arrangements, which were significant at the 10% level. The indirect effect of COVID-19 on the intention of having three children through the influence of childcare arrangement accounted for 6.5% of the total effect.

As shown in model 2, COVID-19 had negative indirect effects on the intention of three children through increased parenting childcare costs, which were significant at the 1% level. The indirect effect of COVID-19 on the intention of having three children through increased childcare costs accounted for 5.9% of the total effect.

## Discussion

Research on the impact of the COVID-19 pandemic on the social, economic, and demographic fields has become a major topic of concern in recent years. This paper concludes that despite the opening of the three-child policy, people's fertility intentions remain low due to the influence of COVID-19. Among them, women are more likely to maintain a negative attitude toward having three children. And the number of existing children will also affect Chinese residents' intention to have a third child.

In previous studies, there were many internal reasons for women's reluctance to have children, including age, annual family income, household registration type, spouse's share of housework, and women's work-childbearing conflict [48–51], the material support of elders and the support of taking care of children, the subjective and objective social stratification of women and the traditional gender concept [54–56]. These factors can significantly affect women's fertility intentions and decisions [52, 57, 58]. In particular, the "de-commercialization" of China's social welfare policies is weak, and women still need to bear the double time squeeze from family and work in terms of fertility care and welfare, which leads to women being more cautious about having multiple children. The findings of lower female intention to have three children obtained in this paper coincide with some of the existing studies. They believe that women are more reluctant to have children than men under the two-child or three-child policy [59–61]. As the primary bearer of fertility responsibility, women in the family always have more to give than men.

According to the results, the more children there have, the more willing they are to have three children [62, 63]. According to existing studies, the positive result between the number of children and the intention to have children can be explained by the Leibenstein-style marginal utility difference of children: if the number of children increases within a certain range [53], the cost of childbearing may decrease [64]. In addition, giving birth to a third child is different from giving birth to a first child or a second child [65–67]. In the absence of gender preference, the substitution effect is usually insignificant for parents who give birth to a third child [68, 69]. For instance, Career Women choose to give up their jobs and take care of their children full-time at home after having their first child. Under these conditions, the opportunity cost of having a second child will be significantly lower than that of having a first child. Moreover, the benefits that children bring to the family, such as family happiness, are likely to multiply. Therefore, the marginal cost of childbearing within a certain range decreases while the marginal utility increases. Thus, having children brings happiness to parents on a certain level. The marginal utility of having another child in a family that has already had a child is less than in a family that has not had a child. Under the dual influence of subjective happiness view and fertility marginal effect, Chinese residents with 1 child, and 2 children gradually increase their intention to have a third child.

Parenting is a long and complicated process. Giving birth during the COVID-19 pandemic will face numerous potential dangers and challenges. Yue J and Fan X highlight that due to the economic and social changes in recent years, the work intensity of parents in the family, the refinement level of childcare, and the cost of childcare have increased simultaneously [70]. As an important stronghold for residents to resist the spread of the virus during the outbreak, families bear more functions but also contain more conflicts. This conflict is most significant in childcare activities and housework. At the same time, families, schools, and society share the responsibility for childcare. However, some of the child-care responsibilities that would have been borne by schools and society under the ongoing suspension of schools following the outbreak have been added to families. Research-based on the data of the China labor Dynamics Survey (CLDS) shows that childcare is a key constraint on family fertility intention.

Therefore, this paper argues that due to the impact of COVID-19 and the restrictions of pandemic control policies, Chinese residents have been affected in their childcare arrangements. This change in childcare arrangements makes residents have more difficulties and problems in childcare activities, which further affects residents' intention to have children again.

During the COVID-19 pandemic, social production has been hampered, resulting in a rise in the cost of all kinds of life products. The cost of childcare for US families during COVID-19 is on average 47% higher than the cost of pre-COVID-19 care [71]. In general, the rational choice of family members in making fertility decisions will consider the various costs and pressures of childcare. The cost of rearing is still the main factor affecting female fertility [72]. In terms of the needs of different children, In China, Scholars in their research found the intention to have a second child that the cost of rearing is the main factor affecting whether parents of child-bearing age had a second child or not. Then, they put forward that Chinese residents' intention to have a third child is low, which is generally affected by economic costs and time costs.

Combined with the results of the model, the authors believe that due to the outbreak of COVID-19, families have to meet their safety and health needs and cope with the income decline caused by school suspension, which directly leads to an increase in family childcare costs. Whether it is the economic cost or psychological cost, explicit cost or implicit cost, direct cost or indirect cost, the increase in childcare costs will lead to a decline in residents' intention to bear children.

Under the influence of anti-pandemic actions and control policies, COVID-19 pandemic has different impacts on different occupational groups, among which medical workers and transportation service workers are most affected [73]. Due to the nature of the work of these two groups, they have to be highly exposed to the virus to fight against the pandemic and maintain the normal life of residents, which makes the infection rate of the virus in occupational groups significantly higher than that in other professions [74]. According to statistics by Fan and Yang, et al., the detection rate of psychological problems among medical staff during the period of COVID-19 pandemic has been as high as 39.3% [75].

From a micro perspective, the medical staff, especially female staff, had negative emotions toward fertility to some extent during the COVID-19 pandemic through qualitative research [76]. The negative emotions are mainly caused by fears of infection and the effects of heavy use of sterilizing drugs (e.g., ethanol) on their fertility. In addition, service occupations also receive a big impact. Such people need to take on more of the risk of COVID-19 pandemic. COVID-19 has also led to a continuous downturn in the job market of civil aviation and transportation, which has increased the job risks of those engaged in high-speed rail and bullet trains related to aviation, and a grim employment situation for students majoring in this field.

Therefore, the author believes that the exposure risk of COVID-19 in certain occupational groups is higher than that of other groups, and the threat of the risk of COVID-19 pandemic will cause residents in these occupational groups to temporarily reduce their intention to have children and delay their family planning. In the descriptive statistics, the employment personnel with two high risks accounts for 31.11% of the total number, which also strongly supports the empirical data results and the overall trend of fertility intention slightly decreased.

In conclusion, this paper holds that the pandemic further inhibits residents' intention to have a third child by affecting their childcare arrangements, increasing their childcare costs, and their occupational exposure to the virus, which is valid.

## Limitations and future research directions

This study provides fresh evidence and an important glimpse of the possible impact of COVID-19 on fertility intention. However, it still has several limitations.

First, this study is based on a web-based questionnaire collected by a self-administered survey, and it does not cover all regions of China, nor does it include all potential populations. But this is probably the best way to investigate the state of epidemic prevention and control. The current COVID-19 policy in China has changed considerably, and we expect subsequent scholars to conduct more related surveys with a wider scope, fuller samples, and better representativeness. We expect scholars to obtain different results in this way to promote the diversity of related studies.

Second, due to the limitation of data, a simple "stratification + summation" method is used to measure the impact of the COVID-19 epidemic in this paper. Although this approach has a theoretical and practical basis, it is more or less deficient in its logical derivation and formation of pathways. Measuring the impact of the epidemic is a systematic and large topic, and if we would like more diversity in their subsequent studies, then identifying a widely recognized indicator for measuring COVID-19 (to a certain group or individual) will be an important consideration for subsequent scholars' research.

Third, in the mediating effects section, for the effects of COVID-19 on people's childcare arrangements and childcare costs we can only analyze them based on respondents' yes/no responses, which does not allow us to obtain a statistically significant proportion or value to estimate how much of a disturbance is present. The impact of COVID-19 on people's lives is multifaceted, at this study the dichotomous variables may yield intuitive results more rapidly, but this intuition comes at the cost of ignoring differences in cognitive criteria. We expect subsequent studies can consider more measurable policy factors, willingness factors, environmental factors, etc. in their studies. Despite these limitations, our study still provides additional evidence on the mechanisms by which catastrophes with broad effects, such as COVID-19, affect people's willingness to have children. Although our study is country-specific, we believe it can still provide some insight into the critical issues faced and help make the right decisions and choices in areas of need.

Fourth, in the linear regression model, R2 indicates the goodness of fit, and the larger the goodness of fit (maximum value 1), the higher the degree of explanation of the dependent variable by the independent variable. In the model results of this paper, the R2 of the model without the inclusion of control variables is only 0.033 and with the inclusion of control variables it is 0.085. Although in many existing studies, as a regression analysis model (rather than a prediction model), R2 measures the variance of the unobserved component versus the observed component and is relevant to whether our point of interest lies in the effect of x on y. But, in any case, the low R2 value is always an unavoidable limitation of this paper. Although it only responds to the degree of model fit, and a lower value can occur in the field of empirical research where the research perspective is relatively novel, there is no denying its current status of poor performance in terms of how well the sample regression fits the data, and we do not deny it and would like to share our research and the problems encountered with all scholars to discuss a unified opinion that is widely accepted.

Finally, due to data access, we were unable to obtain data on the geographical location of the respondents for model analysis. However, in fact, if we could obtain spatial fixed effects or exogenous variables at the district level through spatial location, it would make the model setting more relevant to the real situation. In addition, it is also very meaningful to analyze the differences in the intention to have three children among residents in different regions.

## Conclusions

Based on the empirical analysis, this study found that gender and the number of children will affect the residents' intention to have a third child. Despite the opening of the three-child policy, people's willingness to have three children remains low under the influence of COVID-19, and among them, women are more resistant to having three children than men. The effect of the three-child policy on fertility in China may be less effective the impact of COVID-19. We investigated the mechanism and concluded that the increased cost of childcare during COVID-19, the impact on childcare arrangements, and the greater risk of viral infection in some occupations interfere with people's willingness to have three children.

At present, the development of COVID-19 in China has basically stabilized, and people's lives have generally recovered as before under a more relaxed control policy, and along with the implementation of supporting measures to encourage the birth of three children, it is almost foreseeable that the subsequent fertility rate of two and three children in China will increase again. Perhaps the policy effect of the three-child policy will start to show its effect and play its original value in the coming period.

### Prospects

At present, China's three-child policy is still in the stage of continuous improvement and development, and the outbreak of COVID-19 is an important factor hindering the implementation of the three-child policy. Lessons learned during COVID-19 will form an important basis for developing relevant policy measures and limiting the adverse impact of similar events in the future. Therefore, this paper will put forward some prospects to provide a reference for relevant research and provide a basis for China and other countries to formulate post-PANDEMIC fertility policies and improve the birth security system.

First, the inhibition effect of major disasters on fertility intention and fertility behavior will be significant in the early stage of the disaster but will recover somewhat in the middle and late stage. Those family planning programs that were postponed or suspended due to the pandemic may be resumed when the situation is stable, policy support, and comprehensive security are provided, showing a resurgence similar to "compensatory" birth. Combined with the successful issuance of follow-up guarantee policies of the three-child policy, this paper holds that the emergence of the next "inflection point" of birth in China is not far away. The premise is to face up to the pain point of Chinese residents at the present stage and predict the possible itchy point of fertility in the future.

Second, focus on the analysis of fertility confusion in different groups during the pandemic. Women of childbearing age are at risk of being exposed to infection, and repeated outbreaks and the threat of malignant consequences may lead to varying degrees of negative emotions among residents, which may affect their family planning. At the same time, high-risk occupational groups under the pandemic are faced with more occupation-attached virus infection threats, and their family planning and intention to have children will be delayed or temporarily suspended due to realistic work pressure and risk exposure anxiety. How to provide solutions for these groups should be the focus of attention.

Finally, fertility intentions are only a tendency and do not necessarily translate into fertility behavior [77]. The gap between intentions and behaviors has become a regular phenomenon in developed and developing countries [78, 79]. Therefore, more research and initiatives to enhance fertility intentions and remove the conversion of fertility intentions into actual fertility behavior should be proposed.

## Data Availability

The data used in this paper is a brand new one. After being collected and processed, the empirical analysis is carried out for the first time and the data results are tried to be published. Due to [relevant data protection laws] the datasets are available only to the collaborating scientists from the Population Policy and Development Research Center of Chongqing Technology and Business University (PDPR-CTBU) or China Population and Development Research Center (CPDRC) but are available from the first author (Prof. Zi Li) on reasonable request. At present, the dataset exists in a private way, and no accessible links have been published, but it is not ruled out that it may be disclosed to the outside world after some time. (Official website of PDPR-CTBU https://cpdp.ctbu.edu.cn/plistx.aspx?cid=11&c=&pid=4).
